# The association of serum irisin with anthropometric, metabolic, and bone parameters in obese children and adolescents

**DOI:** 10.3389/fendo.2023.1326851

**Published:** 2024-01-25

**Authors:** Shin-Hee Kim, Sung Eun Kim, Seulki Kim, Moon Bae Ahn, Won Kyoung Cho, Kyoung Soon Cho, Min Ho Jung

**Affiliations:** ^1^ Department of Pediatrics, Incheon St. Mary’s Hospital, College of Medicine, The Catholic University of Korea, Seoul, Republic of Korea; ^2^ Department of Pediatrics, Eunpyeong St. Mary’s Hospital, College of Medicine, The Catholic University of Korea, Seoul, Republic of Korea; ^3^ Department of Pediatrics, Seoul St. Mary’s Hospital, College of Medicine, The Catholic University of Korea, Seoul, Republic of Korea; ^4^ Department of Pediatrics, St. Vincent's Hospital, College of Medicine, The Catholic University of Korea, Seoul, Republic of Korea; ^5^ Department of Pediatrics, Bucheon St. Mary’s Hospital, College of Medicine, The Catholic University of Korea, Seoul, Republic of Korea; ^6^ Department of Pediatrics, Yeouido St. Mary’s Hospital, College of Medicine, The Catholic University of Korea, Seoul, Republic of Korea

**Keywords:** irisin, obesity, insulin resistance, bone mineral density, children

## Abstract

**Background:**

Irisin is an adipomyokine secreted by muscle and adipose cells, and it plays a role in glucose, fat, and bone metabolism. This study aimed to determine the correlation of serum irisin levels with anthropometric, metabolic, and bone parameters in obese children and adolescents.

**Methods:**

This single-center study included 103 Korean children and adolescents: 54 (52.4%) obese participants with a body mass index (BMI) ≥95th percentile and 49 (47.6%) healthy controls with BMI within the 15th to 85th percentile. Various parameters were measured, including fasting blood glucose, fasting insulin, homeostasis model assessment of insulin resistance (HOMA-IR), triglyceride and glucose (TyG) index, lipid profile, alkaline phosphatase (ALP), osteocalcin, and 25(OH)-Vitamin D levels. Bone mineral density (BMD) was measured using dual-energy X-ray absorptiometry (DEXA) in 33 healthy subjects.

**Results:**

Serum irisin was significantly higher in the obese group than in the control group (mean 18.1 ± 3.5 vs. 16.2 ± 2.0 ng/mL; *p* = 0.001). Serum irisin level was positively correlated with chronological age (*r* = 0.28; *p* = 0.004), height SDS (*r* = 0.24; *p* = 0.02), BMI SDS (*r* = 0.37; *p* < 0. 001), fasting glucose (*r* = 0.27; *p* = 0.007), fasting insulin (*r* = 0.23; *p* = 0.03), HOMA-IR (*r* = 0.21; *p* = 0.04), osteocalcin (*r* = 0.27; *p* = 0.006) and negatively correlated with HDL cholesterol (*r* = -0.29; *p* = 0.005). All these correlations were evident in obese subjects but not in healthy subjects. ALP and 25(OH)-Vitamin D were unrelated to irisin levels. Among 33 healthy subjects, total body-less head (TBLH) BMD Z-score was positively correlated with serum irisin (*r* = 0.39; *p* = 0.03), osteocalcin (*r* = 0.40; *p* = 0.02), fasting insulin (*r* = 0.39; *p* = 0.04), and HOMA-IR (*r* = 0.38; *p* = 0.047).

**Conclusion:**

This study demonstrated an association between irisin levels and glucose, lipid, and bone parameters in children and adolescents. Our findings suggest that irisin has a potential role in metabolic disorders and bone health in obese children and adolescents.

## Introduction

Muscle cells produce and release cytokines and chemokines, collectively known as myokines, during physical activity. Representative myokines include myostatin, brain-derived neurotrophic factor (BDNF), interleukin-6, fibroblast growth factor-21 (FGF-21), monocyte chemotactic protein 1 (MCP1), insulin-like growth factor-1 (IGF-1), and irisin (1). Irisin was first identified as a myokine secreted by muscle in response to exercise (2), later determined to be also secreted by white adipose tissue (3). Irisin is cleaved from its precursor fibronectin type III domain-containing protein 5 (FNDC5) in skeletal muscle cells and is responsible for white fat browning, promoting thermogenesis and energy expenditure (4).

Irisin facilitates glucose uptake by skeletal muscles and improves glucose and lipid profile levels in obesity and type 2 diabetes patients, making it an important factor in metabolic regulation (5). Circulating irisin was significantly lower in individuals with T2DM than in non-diabetic controls (6). In non-diabetic patients, circulating irisin levels correlate positively with fasting glucose, fasting insulin, and homeostatic model assessment (HOMA) (6, 7). Irisin has recently received much attention in the treatment of obesity, which is one of the biggest public health problems worldwide. Animal studies have shown downregulation of FNDC5 in both skeletal muscle and adipose tissue due to obesity (8, 9), while others have shown no significant association between obesity and FNDC5/irisin (10, 11). In humans, some studies showed a positive correlation between serum irisin levels and body mass index (BMI) (6, 7), but others found negative (12) or no association (13).

Recent evidence suggests that irisin also plays an important role in bone metabolism and health (14). *In vitro* experiments, irisin promotes osteoblast proliferation and differentiation via activating mitogen-activated protein (MAP) kinase signaling pathways (15). Colaianni et al. found that recombinant irisin increased cortical bone content and prevented bone loss in mice (16). Cortical and trabecular bone mineral density (BMD), bone volume fraction loss, and fractal dimension are preserved in irisin-treated mice subjected to musculoskeletal unloading (17). In humans, irisin levels positively correlated with BMD and strength in soccer players (18).

Despite these concerns, limited studies have addressed the relationship between irisin and obesity and metabolic parameters in children and adolescents (19–22). Obesity is a growing health threat in children and adolescents, and irisin has therapeutic potential. Furthermore, scarce data exist on the relationship between irisin and bone status in this population (23, 24). Therefore, our study aimed to investigate the relationship of serum irisin levels with anthropometric, metabolic, and bone parameters in Korean children and adolescents.

## Materials and methods

### Subjects

This study was conducted with 103 children and adolescent patients who visited the outpatient Department of Pediatrics at Incheon St. Mary’s Hospital from March 2020 to August 2021. Based on Korean children and adolescent growth charts in 2017, the standard deviation score (SDS) of height, weight, and BMI was calculated for gender and age (25). Obesity was defined as a BMI greater than or equal to the 95th percentile. The normal weight was defined as a BMI between the 15th and 84th percentile of the same age and gender. Exclusion criteria included children with chronic disease (liver disease, renal disease), genetic disorders (Turner syndrome, Prader-Willi syndrome, Congenital adrenal hypoplasia), and endocrine disorders (thyroid disease, Cushing disease, Type 2 diabetes mellitus). Written informed consent was obtained from all participants and their parents. The study was approved by the Institutional Review Board of Incheon St. Mary’s Hospital (IRB number: OC19OESI0063, OC21OESI0119).

### Anthropometry measurements

Height was recorded to the nearest 0.1 cm using a Harpenden Stadiometer, and weight was recorded to the nearest 0.1 kg using an electronic scale. Weight, height, and BMI were not evenly distributed among the different age groups, and the standard deviation scores (SDS) were based on the reference data published by the 2017 Korean National Growth Charts (25).

The pubertal stage was assessed by the pediatric endocrinologist according to Marshall and Tanner standards (26). The pubertal stage was defined as testicular volume ≥4 mL in boys and breast development in girls. Tanner stage 1 was defined as prepuberty, Tanner stage 2–3 as early puberty, and Tanner stage 4–5 as late puberty.

### Laboratory evaluations

Venous blood samples were obtained in the morning after a 10-hour overnight fast. Serum samples were analyzed for glucose, insulin, total cholesterol (TC), triglyceride (TG), high-density lipoprotein cholesterol (HDL-C), low-density lipoprotein cholesterol (LDL-C), alkaline phosphatase (ALP), 25(OH)-Vitamin D, osteocalcin, and irisin. Dyslipidemia was defined as TC ≥200 mg/dL, LDL-C ≥ 130 mg/dL, HDL-C ≤ 45 mg/dL, and TG ≥150 mg/dL (or a combination thereof) according to the Third Report of the National Cholesterol Education Program (27) and the American Diabetes Association (28). Insulin resistance was estimated from fasting glucose and insulin serum levels using the homeostasis model assessment of insulin resistance (HOMA-IR): fasting insulin (µU/mL)×fasting glucose (mmol/L)/22.5. Serum osteocalcin was measured using an electrochemiluminescence immunoassay (ECLIA) and an Elecsys autoanalyzer (Roche Diagnostics GmbH, Mannheim, Germany). Serum irisin levels were measured using an ELISA assay (Phoenix Pharmaceuticals, Burlingame, CA). The intra- and inter-assay coefficients of variation (CV) were 4–8% and 8–12%, respectively.

### Dual-energy X-ray absorptiometry

BMD was measured in the total body-less head region (TBLH) using a Lunar model DXA (Lunar Prodigy Advance; GE Healthcare Lunar Corporation, Madison, WI, USA). BMD SDS (TBLH Z-score) refers to the standard value of BMD in Korean children and adolescents (29). Due to a limited research budget, BMD was measured only in healthy subjects but not in obese subjects. We have selected healthy rather than obese subjects for BMD measurement to exclude the confounding effects of metabolic parameters associated with obesity.

### Statistical analysis

All statistical analyses were conducted using SPSS for Windows version 20.0 (SPSS Inc., Chicago, IL, USA). Chi-square tests or Fisher’s exact tests were employed for categorical variables between two groups, as appropriate. The Student’s t-test and Mann-Whitney U test were utilized for continuous variables with normal and non-normal distributions, respectively. The normality of distribution was assessed using the Shapiro-Wilk test. The correlation between continuous variables was examined using Pearson’s correlation coefficient analysis.

Multiple linear regression was used to estimate the independent association between irisin levels and metabolic parameters. All p-values < 0.05 were considered statistically significant.

## Results

### Clinical characteristics of obese and normal weight groups

Of all subjects, 54 (52.4%) were obese and 49 (47.6%) were normal weight. **Table 1** shows the clinical and biochemical characteristics in the obese and normal weight groups. The mean age of the obese and normal weight groups was 10.2 ± 2.4 years and 9.8 ± 1.7 years, respectively. Median BMI was 25.3 kg/m^2^ in the obese group and 18.1 kg/m^2^ in the normal weight group (*p* < 0.001). Dyslipidemia was more common in the obese group than in the normal weight group (72.2% vs. 40.8%; *p* = 0.001).

**Table 1:** Demographic, clinical, and laboratory data for obese and control subjects.

Characteristic	Obese (n = 54)	Control (n = 49)	*p-*value
Chronological age (years)	10.2 ± 2.4	9.8 ± 1.7	0.33
Sex			0.73
Male, n (%)	25 (46.3)	21 (42.9)	
Female, n (%)	29 (53.7)	28 (57.1)	
Height SDS	0.8 ± 1.2	0.4 ± 1.3	0.15
Weight SDS	2.1 ± 0.6	0.3 ± 0.8	<0.001
BMI (kg/m^2^)	25.3 (23.7–27.9)	18.1 (17.1–19.0)	<0.001
BMI SDS	2.3 (2.1–2.6)	0.2 (-0.1–0.8)	<0.001
Pubertal stage			0.30
Prepurberty, n (%)	26 (48.1)	21 (42.9)	
Early puberty, n (%)	17 (31.5)	22 (44.9)	
Late puberty, n (%)	11 (20.4)	6 (12.2)	
Fasting glucose, mg/dL	93.0 (87.0–103.5)	93.0 (87.5–99.5)	0.79
Fasting insulin, μU/mL	22.7 (15.3–32.0)	22.0 (14.3–32.0)	0.83
HOMA-IR	5.5 (3.7–8.1)	5.1 (3.1–7.6)	0.81
Dyslipidemia, n (%)	39 (72.2)	20 (40.8)	0.001
Total cholesterol (mg/dL)	172.6 ± 20.7	163.7 ± 25.8	0.15
LDL cholesterol (mgL)	108.0 ± 16.3	102.2 ± 16.8	0.09
HDL cholesterol (mg/dL)	52.8 ± 12.7	55.8 ± 7.6	0.18
Triglycerides (mg/dL)	146.0 (100.0–175.3)	117.5 (92.3–153.0)	0.06
TyG index	8.8 (8.5–9.1)	8.7 (8.4–9.0)	0.19
ALP (U/L)	91.5 ± 20.8	99.6 ± 24.2	0.07
Osteocalcin (ng/mL)	81.4 (66.9–99.2)	78.9 (67.0–91.4)	0.75
25(OH)-Vitamin D (ng/mL)	14.4 ± 4.8	14.0 ± 4.4	0.71
Irisin, ng/mL	18.1 ± 3.5	16.2 ± 2.0	0.001

Data are presented as the mean ± standard deviation for continuous parametric variables or as the median (interquartile range) for continuous nonparametric variables unless otherwise specified. ALP, alkaline phosphatase; BMI, body mass index; HDL, high-density lipoprotein; HOMA-IR, homeostasis model assessment-insulin resistance; LDL, low-density lipoprotein; SDS, standard deviation score; TyG index, triglyceride and glucose index.

### Correlation of serum irisin with patient characteristics

**Figure 1:**
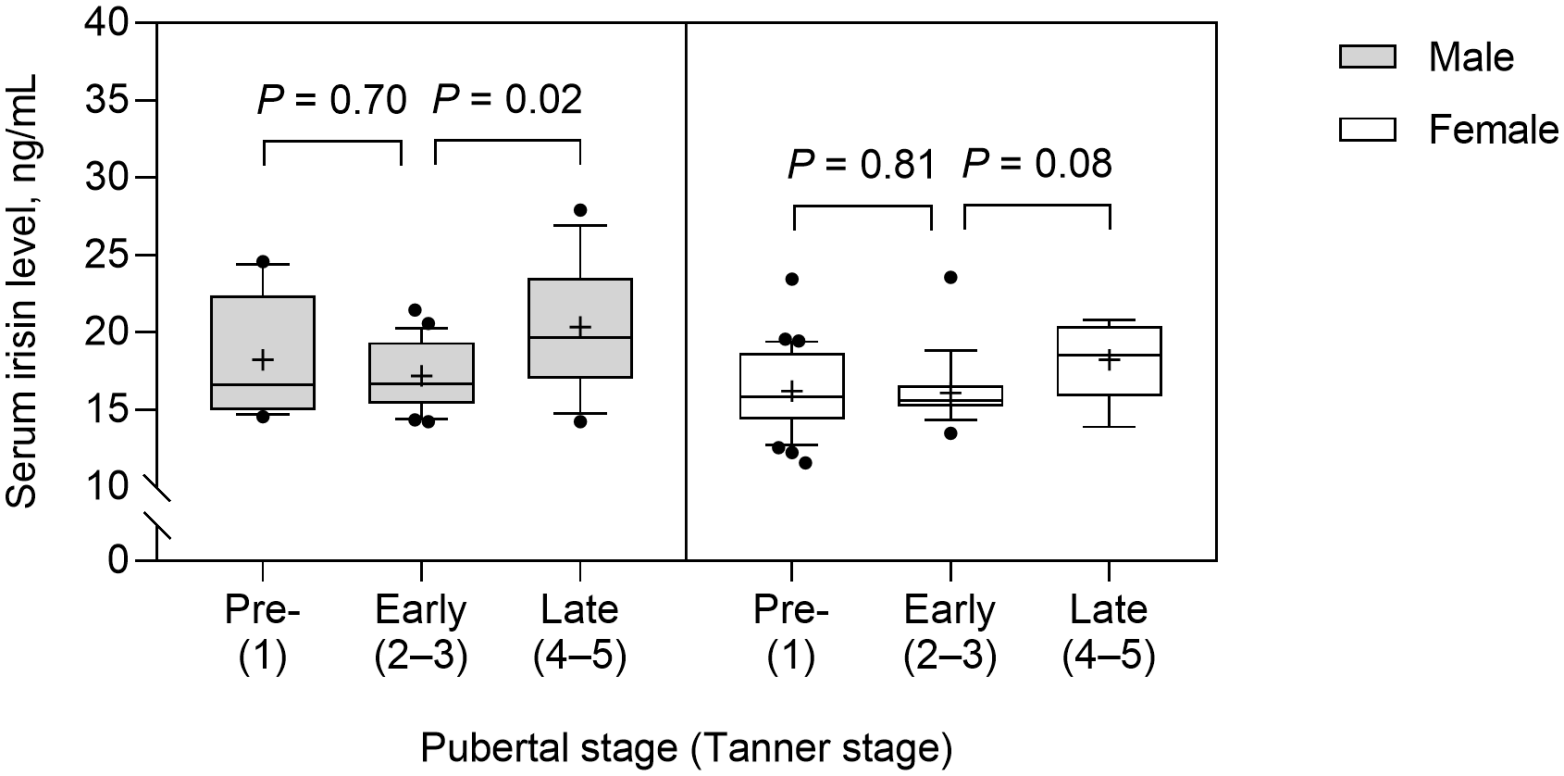
Serum irisin levels according to the pubertal stage in male and female subjects. The box and whisker plots indicate the median (horizontal line in the box), 25^th^ percentile (bottom line of the box), 75^th^ percentile (top line of the box), and 5^th^ and 95^th^ percentiles (whiskers). The black cross and dots indicate the mean and outliers, respectively.

Serum irisin level was significantly higher in boys than in girls (median 17.5 vs. 15.8; *p* = 0.003) and in the obese group than in the control group (mean 18.1 ± 3.5 vs. 16.2 ± 2.0 ng/mL; *p* = 0.001). Serum irisin levels were not significantly different between prepubertal and early pubertal children. However, serum irisin levels were significantly higher in late puberty than early puberty (median 19.6 vs. 16.7; *p* = 0.02) in boys, but this difference did not reach a significant level in girls (median 18.5 vs. 15.4; *p* = 0.08) (**Figure 1**). The subjects with dyslipidemia had significantly higher serum irisin levels than those without (mean 17.8 ± 3.3 vs. 16.4 ± 2.4 ng/mL; *p* = 0.01).

### Correlation of serum irisin with anthropometric and metabolic parameters

**Table 2** shows the correlation between serum irisin and anthropometric and metabolic parameters. Serum irisin level was positively correlated with chronological age (*r* = 0.28; *p* = 0.004), height SDS (*r* = 0.24; *p* = 0.02), weight SDS (*r* = 0.41; *p* < 0.001), BMI SDS (*r* = 0.37; *p* < 0. 001), fasting glucose (*r* = 0.27; *p* = 0.007), and fasting insulin (*r* = 0.23; *p* = 0.03), HOMA-IR (*r* = 0.21; *p* = 0.04). Serum irisin level negatively correlated with HDL cholesterol (*r* = -0.29; *p* = 0.005). All these correlations were evident in obese subjects but not in healthy subjects. **Table 3** shows multiple linear regression analysis between serum irisin levels and metabolic parameters. Multiple linear regression analysis indicated that BMI SDS was independently associated with serum irisin (β = 0.87; *p* = 0.002) in all subjects. In the obese group, BMI SDS (β = 2.90; *p* = 0.03) and HOMA-IR (β = 0.33; *p* = 0.03) were independently associated with serum irisin levels. In the healthy group, no factors were associated with serum irisin levels.

**Table 2:** Correlation of serum irisin with anthropometric, metabolic, and bone parameters.

Characteristic	All subjects (n = 103)		Obese (n = 54)		Control (n = 49)	
	*r*	*p*-value	*r*	*p*-value	*r*	*p*-value
Chronological age (years)	0.28	0.004	0.27	0.046	0.25	0.08
Height SDS	0.24	0.02	0.43	0.001	-0.15	0.30
Weight SDS	0.41	<0.001	0.46	0.001	0.11	0.47
BMI (kg/m^2^)	0.49	<0.001	0.49	<0.001	0.32	0.02
BMI SDS	0.37	<0.001	0.39	0.004	0.11	0.44
Fasting glucose, mg/dL	0.27	0.007	0.29	0.04	0.22	0.14
Fasting insulin, μU/mL	0.23	0.03	0.28	0.046	0.20	0.19
HOMA-IR	0.21	0.04	0.33	0.02	0.16	0.30
Total cholesterol (mg/dL)	0.19	0.05	0.15	0.27	0.17	0.25
LDL cholesterol (mgL)	0.15	0.15	0.15	0.30	0.04	0.82
HDL cholesterol (mg/dL)	-0.29	0.005	-0.34	0.01	0.04	0.79
Triglycerides (mg/dL)	0.19	0.05	0.20	0.16	0.07	0.62
TyG index	0.08	0.46	0.27	0.06	-0.09	0.57
ALP, U/L	-0.05	0.60	-0.07	0.64	0.12	0.42
Osteocalcin (ng/mL)	0.27	0.006	0.42	0.002	0.06	0.67
25(OH)-Vitamin D (ng/mL)	0.06	0.60	0.01	0.95	0.13	0.43

ALP, alkaline phosphatase; BMI, body mass index; HDL, high-density lipoprotein; HOMA-IR, homeostasis model assessment-insulin resistance; LDL, low-density lipoprotein; SDS, standard deviation score; TyG index, triglyceride and glucose index.

**Table 3:** Multiple linear regression analysis between serum irisin level and metabolic parameters.

Characteristic	All subjects (n = 103)		Obese (n = 54)		Control (n = 49)	
	β	*p*-value	β	*p*-value	β	*p*-value
BMI SDS^a^	0.87	0.002	2.90	0.03	0.74	0.26
HOMA-IR^b^	0.15	0.09	0.33	0.03	0.07	0.42
HDL cholesterol^c^	-0.06	0.054	-0.06	0.11	0.03	0.51

BMI SDS, body mass index standard deviation score; HOMA-IR, homeostasis model assessment-insulin resistance; HDL, high-density lipoprotein. Fasting insulin and fasting glucose were not included in the multiple linear regression model due to its multicollinearity with HOMA-IR.

### Correlation of serum irisin with bone parameters and BMD

The correlation of serum irisin with bone parameters is shown in **Table 2**. Serum irisin level was positively associated with osteocalcin (*r* = 0.27; *p* = 0.006). This association was evident in obese subjects (*r* = 0.42; *p* = 0.002) but not in healthy subjects (*r* = 0.06; *p* = 0.67). Serum irisin levels were not associated with ALP (*r* = -0.05; *p* = 0.60) and 25(OH)-Vitamin D (*r* = 0.06; *p* = 0.60).

Of 49 healthy subjects, 33 (67.3%) agreed to the BMD measurement. There were no significant differences in parameters except for weight SDS (*p* = 0.04) and LDL cholesterol (*p* = 0.03) between subjects who received BMD measurement and those who did not (**Table 4**). **Table 5** shows the correlation of TBLH BMD Z-score with anthropometric, metabolic, and bone parameters. The TBLH BMD Z-score was positively correlated with fasting insulin (*r* = 0.39; *p* = 0.04), HOMA-IR (*r* = 0.38; *p* = 0.047), osteocalcin (*r* = 0.40; *p* = 0.02), and serum irisin (*r* = 0.39; *p* = 0.03). ALP and 25(OH)-Vitamin D was not associated with TBLH BMD Z-score.

**Table 4:** Comparison between healthy subjects who underwent bone mineral density measurement and those who did not.

Characteristic	BMD measurement (n = 33)	No BMD measurement (n = 16)	*p-*value
Chronological age (years)	9.5 ± 1.1	10.4 ± 2.3	0.16
Male sex, n (%)	12 (36.4)	9 (56.3)	0.19
Height SDS	0.4 (-0.5–1.5)	-0.2 (-1.0–1.1)	0.39
Weight SDS	0.5 ± 0.7	0.0 ± 0.9	0.04
BMI (kg/m^2^)	18.2 ± 1.3	17.7 ± 1.8	0.27
BMI SDS	0.5 (0.1–0.8)	0 (-0.4–0.2)	0.003
Fasting glucose, mg/dL	94.0 ± 10.4	93.7 ± 9.0	0.93
Fasting insulin, μU/mL	21.5 (14.5–32.0)	22.5 (14.0–38.6)	0.96
HOMA-IR	5.1 (3.1–7.4)	5.1 (3.2–8.8)	0.91
Dyslipidemia, n (%)	14 (42.4)	6 (37.5)	0.74
Total cholesterol (mg/dL)	165.8 ± 25.2	159.3 ± 27.3	0.41
LDL cholesterol (mgL)	106.3 ± 16.5	94.5 ± 14.8	0.03
HDL cholesterol (mg/dL)	55.3 ± 8.3	56.7 ± 6.1	0.59
Triglycerides (mg/dL)	124.5 (99.3–158.5)	106.5 (83.0–140.0)	0.17
TyG index	8.7 (8.5–9.0)	8.5 (8.2–8.9)	0.21
ALP (U/L)	102.3 ± 25.7	94.1 ± 20.3	0.27
Osteocalcin (ng/mL)	79.2 (71.5–96.4)	73.8 (61.1–89.6)	0.25
25(OH)-Vitamin D (ng/mL)	14.0 ± 4.9	14.1 ± 3.2	0.96
Irisin, ng/mL	16.0 ± 1.9	16.6 ± 2.2	0.32

Data are presented as the mean ± standard deviation for continuous parametric variables or as the median (interquartile range) for continuous nonparametric variables unless otherwise specified. ALP, alkaline phosphatase; BMI, body mass index; HDL, high-density lipoprotein; HOMA-IR, homeostasis model assessment-insulin resistance; LDL, low-density lipoprotein; SDS, standard deviation score; TyG index, triglyceride and glucose index.

**Table 5:** Correlation of TBLH BMD Z-score with anthropometric and metabolic parameters, bone turnover markers, and serum irisin.

Characteritic	*r*	*p*-value
Chronological age (years)	0.33	0.06
Height SDS	0.10	0.60
Weight SDS	0.17	0.35
BMI (kg/m^2^)	0.21	0.23
BMI SDS	0.01	0.97
Fasting glucose, mg/dL	0.07	0.73
Fasting insulin, μU/mL	0.39	0.04
HOMA-IR	0.38	0.047
Total cholesterol (mg/dL)	-0.01	0.94
LDL cholesterol (mgL)	-0.12	0.56
HDL cholesterol (mg/dL)	-0.30	0.13
Triglycerides (mg/dL)	0.14	0.45
TyG index	0.13	0.49
ALP (U/L)	0.24	0.18
Osteocalcin (ng/mL)	0.40	0.02
25(OH)-Vitamin D (ng/mL)	0.23	0.27
Irisin, ng/mL	0.39	0.03

ALP, alkaline phosphatase; BMD, bone mineral density; BMI, body mass index; HDL, high-density lipoprotein; HOMA-IR, homeostasis model assessment-insulin resistance; LDL, low-density lipoprotein; ND, not determined; SDS, standard deviation score; TBLH, total body less head. TyG index, triglyceride and glucose index.

## Discussion

This study explores the correlation of serum irisin levels with anthropometric, metabolic, and bone parameters in Korean children and adolescents. The results indicate that irisin levels were positively correlated with age, BMI, fasting glucose, HOMA-IR, and osteocalcin and negatively correlated with osteocalcin. All these associations were evident in obese subjects but not in healthy subjects. In healthy subjects, a positive correlation was found between serum and BMD.

Consistent with the previous results (19–22), we found that serum irisin levels were higher in obese healthy children and adolescents than in healthy subjects and were positively associated with BMI. Although this finding may be expected given the increase in muscle mass (30), increased secretion from adipose tissue is another possible source of increased circulating irisin in obesity. A molecular animal-based study confirmed the contribution of adipose tissue to circulating irisin levels (31). In rats, secretion of irisin was higher from white adipose tissues of diet-induced obese compared to lean controls (31). A study including 145 female adults reported that both fat mass and fat-free mass positively correlated with irisin levels (32). In adolescents, increased body fat mass rather than BMI was an independent factor for increased irisin levels (21). The results of previous studies and ours suggest that increased irisin may compensate for increasing body mass, especially body fat mass in obese individuals, to maintain the balance of energy storage and expenditure. It is also suggested that increased irisin in obesity may be to overcome insulin and irisin resistance (7), similar to well-documented leptin resistance in obesity (33). Although we have not collected the data on physical activity, the association between irisin and BMI in children and adolescents should be cautiously interpreted according to physical activity. One study showed that adolescents who engaged in regular physical activity had higher levels of irisin than sedentary adolescents (21). Another study found significant interactions between circulating irisin concentrations and physical activity on BMI in children (34).

We found a positive correlation between irisin and glucose and HOMA-IR in children and adolescents. In a large study of 618 Korean adolescents, circulating serum irisin positively correlated with glucose, insulin, and HOMAR-IR (21). Elevated serum irisin was associated with increased risk odds of having obesity (OR = 2.2) and metabolic syndrome (OR = 2.0) (21). However, different results were reported in children of different ethnicities and ages. In a cohort study of 153 Saudi children, circulating irisin negatively correlated with HOMA-IR (35). In prepubertal children, circulating negatively correlated with fasting glucose, and children with metabolic syndrome exhibited lower irisin concentrations than those without (36). Notably, the significant association between irisin and HOMA-IR was not observed after controlling for BMI in the previous study (21, 35) and ours. In the pediatric population, serum irisin may be more likely to be strongly associated with body mass and composition than metabolic disease.

In this study, serum irisin levels were negatively correlated with HDL cholesterol and significantly higher in subjects with dyslipidemia. In children, serum irisin negatively correlated with HDL cholesterol (22, 37) and positively correlated with LDL cholesterol (22) and triglyceride (22). One adult study evaluated the association between irisin and lipoprotein subparticles (38). In that study, serum irisin was positively correlated with HDL cholesterol, primarily large HDL particles. Literature suggests a potential role of irisin in cardiac function and cardiovascular diseases. In rats, more irisin is produced in the cardiac muscle than in the skeletal muscle, and serum irisin increased after exercise, higher in younger than older rats (39). In humans, the dysfunction of irisin has been shown to be involved in cardiovascular diseases such as hypertension, coronary artery disease, and myocardial infarction (40). In children, irisin levels were significantly correlated with systolic, diastolic blood pressure, and circulating endothelial progenitor cell levels (37, 41).

We found serum irisin levels positively correlated with osteocalcin in children and adolescents. This finding was consistent with a recent study evaluating 34 healthy children (24). ALP, another osteoblast differentiation marker, did not correlate with serum irisin in the previous study (24) and ours. We found no significant association between irisin and 25(OH)-Vitamin D in children and adolescents. A negative association between irisin and 25(OH)-Vitamin D was reported in children with type 1 diabetes mellitus (42) and Charcot-Marie-Tooth disease (43). In contrast, another study showed that vitamin D supplementation in patients with hypovitaminosis associated with primary hyperparathyroidism increased serum irisin levels (44). Based on these findings, disease type may influence the relationship between irisin and vitamin D. In this study, BMD positively correlated with serum irisin but did not correlate with ALP. Our result was consistent with two recent studies in healthy children of 6–8 years (23) and those of 7–13 years (24). Multivariate regression indicated that irisin was the more powerful determinant of bone mineral status than bone ALP (24). In post-menopausal women, it is negatively associated with vertebral fragility fractures (45, 46). In mice, exercise increased the expression of FNDC5/irisin and osteocalcin in bone tissues (47). Overall, these results suggest that encouraging children to exercise may preserve their bone health, and this could also apply to pediatric pathologic conditions at high risk of poor bone health (48).

Irisin plays an important role in activating the hypothalamic-pituitary-gonadal axis (HPG axis) and in reproductive development. When comparing prepubertal and pubertal children, irisin was higher in pubertal children (37), but other studies have found no difference in irisin levels (21). In this study, there was no significant difference in irisin levels between prepubertal and early pubertal subjects, but irisin levels were higher in late puberty relative to early puberty in boys. Irisin can vary with the ratio of body fat to muscle mass, which varies with sex and pubertal stage, and this should be taken into account when examining changes in irisin levels during puberty (49).

This study has several limitations. First, this is a single-hospital study with a small number of subjects. Thus, this study had limited power to confirm the independent association between irisin and other parameters in obese and healthy subgroups. Second, due to a limited research budget, we have measured BMD in only healthy subjects. Third, body fat mass, muscle mass, diet, exercise, and weight change status were unknown, and the effect of these factors on irisin level remains unclear.

In conclusion, serum irisin levels were associated with BMI, glucose, lipid, and bone parameters in Korean adolescents and children. Our observation is evident in obese subjects but not in healthy subjects. We suggest that irisin is important in regulating glucose, lipid, and bone metabolism in children and adolescents, especially in obese subjects. We found a positive correlation between irisin and BMD in healthy subjects. The potential role of irisin should be confirmed in further studies evaluating the impact of exercise and lifestyle change in obesity on circulating irisin levels, metabolic parameters, and bone health.

## Data availability statement

The original contributions presented in the study are included in the article/supplementary material. Further inquiries can be directed to the corresponding author.

## Ethics statement

The study was approved by the Institutional Review Board of Incheon St. Mary’s Hospital (IRB number: OC19OESI0063, OC21OESI0119). The studies were conducted in accordance with the local legislation and institutional requirements. Written informed consent for participation in this study was provided by the participants’ legal guardians/next of kin.

## Author contributions

S-HK: Data curation, Formal Analysis, Funding acquisition, Writing – original draft, Writing – review & editing, Conceptualization, Investigation, Methodology, Project administration, Resources, Software, Validation, Visualization. SEK: Data curation, Investigation, Writing – review & editing. SK: Methodology, Writing – review & editing, Data curation. MA: Investigation, Writing – review & editing, Methodology. WC: Formal Analysis, Writing – review & editing. KC: Data curation, Writing – review & editing. MJ: Funding acquisition, Supervision, Visualization, Writing – review & editing.
